# Genome-wide association analysis reveals the optimal genomic regions for pod size in bean

**DOI:** 10.3389/fpls.2023.1138988

**Published:** 2023-05-12

**Authors:** Mao Li, Xinyi Wu, Baogen Wang, Xiaohua Wu, Ying Wang, Jian Wang, Junyang Dong, Jian Wu, Zhongfu Lu, Yuyan Sun, Wenqi Dong, Jing Yang, Guojing Li

**Affiliations:** ^1^ Key Laboratory of Quality and Safety Control for Subtropical Fruit and Vegetable, Ministry of Agriculture and Rural Affairs, Collaborative Innovation Center for Efficient and Green Production of Agriculture in Mountainous Areas of Zhejiang Province, College of Horticulture Science, Zhejiang A & F University, Hangzhou, China; ^2^ Institute of Vegetables, Zhejiang Academy of Agricultural Sciences, Hangzhou, China; ^3^ State Key Laboratory for Managing Biotic and Chemical Threats to the Quality and Safety of Agro-products, Zhejiang Academy of Agricultural Sciences, Hangzhou, China; ^4^ Key Laboratory of Vegetable Legumes Germplasm Enhancement and Molecular Breeding in Southern China (Co-construction by Ministry and Province), Ministry of Agriculture and Rural Affairs, Zhejiang Academy of Agricultural Sciences, Hangzhou, China

**Keywords:** pod size, GWAS, KASP marker, quality, snap bean

## Abstract

The snap bean is the most commonly grown vegetable legume worldwide, and its pod size is both an important yield and appearance quality trait. However, the improvement of pod size in snap beans grown in China has been largely hindered by a lack of information on the specific genes that determine pod size. In this study, we identified 88 snap bean accessions and evaluated their pod size traits. Through a genome-wide association study (GWAS), 57 single nucleotide polymorphisms (SNPs) significantly associated with pod size were detected. Candidate gene analysis showed that cytochrome P450 family genes, WRKY, and MYB transcription factors were the predominant candidate genes for pod development, and eight of these 26 candidate genes showed relatively higher expression patterns in flowers and young pods. A significant pod length (PL) SNP and a single pod weight (SPW) SNP were successfully converted into kompetitive allele-specific polymerase chain reaction (KASP) markers and validated in the panel. These results enhance our understanding of the genetic basis of pod size, and also provide genetic resources for the molecular breeding of pod size in snap beans.

## Introduction

The common bean (*Phaseolus vulgaris* L.) (2n = 2x = 22) is the most commonly grown grain and vegetable legume in the world. The common bean belongs to the *Leguminosae* family, which includes tropical legumes such as common beans (*P. vulgaris* L.), cowpeas [*Vigna unguiculata* (L.) Walp.], and soybeans (*Glycine max* L.), and cool season legumes such as peas (*Pisum sativum* L.) and faba beans (*Vicia faba* L.). It was recognized that the common bean was independently domesticated in two regions, now Mexico and South America, and formed the Middle American and Andean gene pools (Schmutz et al., 2014). The cultivated common bean comprised two main types, i.e., dry beans, used for food and fodder, and snap beans, used as vegetables. Dry beans are a valuable source of daily protein and calories globally and are essential to food and nutritional security in developing regions of the world, in particular in African and South American countries (Broughton et al., 2003). Snap bean is the most commonly grown vegetable legume in Europe, North America, and Asia because of its high nutritional content, which includes proteins, vitamins, and minerals (Delfini et al., 2021b).

For snap beans, pod size is not only an important yield-related trait, but also an important commercial trait that determines both consumer acceptance and whether or not good sale prices on the wholesale and retail markets are procured. Similar to other yield-related traits, pod size is a complex quantitative trait that is highly affected by the environment (Campa et al., 2018). Therefore, pod size improvement based on phenotype selection in traditional breeding is both a high-cost and a time-consuming process. To increase the precision and efficiency of selection for these complex traits, using genotype selection by way of marker-assisted tools is the most fundamental strategy, and exploiting the genes controlling the target traits, and developing the linked markers to the target genes, are key steps (Kamfwa et al., 2015). Previous studies have shown that most pod size traits such as pod length (PL), pod thickness (PT), and pod width (PW) displayed quantitative inheritance traits (Yuste-Lisbona et al., 2014; González et al., 2016; Hagerty et al., 2016; Murube et al., 2020), and some pod morphological traits were controlled by several major genes; for example, the cross-sectional shape trait was controlled by at least four genes (i.e., *Ea*, *Eb*, *Ia*, and *Ib*) (Leakey, 1988), the stringless trait was controlled by the *St* gene (Prakken, 1934), the twister trait was controlled by the *Tw* gene (Baggett and Kean, 1995), and the two genes *Da* and *Db* conferred the straight trait (Lamprecht, 1932; Lamprecht, 1947). During the past decade, many quantitative trait loci (QTLs) and SNP loci associated with pod size and pod morphological traits have been reported. Through linkage analysis, Yuste-Lisbona et al. (2014) identified six pod size QTLs on chromosomes Pv01, Pv04, and Pv11 in a recombinant inbred lines (RIL) population, comprising three for PL, two for PW, and one for PT. González et al. (2016) detected five QTLs for pod size on chromosomes Pv01, Pv02, and Pv09 using two RIL populations. Hagerty et al. (2016) mapped four pod size QTLs on chromosomes Pv04 and Pv09 using a RIL population derived from dry beans and snap beans. Murube et al. (2020) detected four QTLs for pod size on chromosomes Pv01, Pv02, Pv07, and Pv11 using two nested RIL populations. Interesting, all the studies have detected some QTLs with pleiotropic effects on PL, PW, and PT, although these were mapped on different chromosomes. Using genome-wide association mapping in a panel of 135 accessions, Gupta et al. (2020) detected two and nine simple sequence repeats (SSRs) associated with PL and PW, respectively. García-Fernández et al. (2021) detected 23 QTLs for PL, six for pod cross-section, and six for pod characters from 301 bean lines of the Spanish Diversity Panel.

The common bean was introduced to China in the 15th century, and the dry bean variety is now among the top six cereal substitute crops, with an annual production of 1.29 million tons. The snap bean is the most commonly grown legume vegetable, with an annual production of 17.99 million tons in China (FAO2020). Over 4,900 common bean accessions have been collected in China, showing notable diversity (Zhang et al., 2008). Wu et al. (2019) have detected one genomic region associated with pod height, 13 genomic regions associated with PL, and 59 genomic regions associated with PW using a diversity panel of 683 common bean accessions, in which most accessions belonged to dry bean. Because of the very limited pod size genes/QTLs reported in Chinese snap bean germplasms, the genetic improvement of pod size in snap bean breeding has been largely hindered in China. To solve this problem, in the present study, we aimed to conduct a genome-wide association study (GWAS) for pod size in a diversity panel of Chinese snap bean accessions, including landraces and cultivars. The results enhance our understanding of the genetic basis of pod size and facilitate the molecular breeding of pod size in snap beans.

## Materials and methods

### Plant materials and pod size evaluation

A diversity panel consisting of 88 snap bean accessions were used in this study. The 88 accessions included 62 landraces and 26 cultivars, of which 12 accessions belonged to the Andean gene pool (Wu et al., 2022). In a previous study, this panel was used to identify the significant genomic regions for bean rust (*Uromyces appendiculatus*) (Wu et al., 2022). All accessions were planted in the Haining Yangdu Scientific Research and Innovation Base of Zhejiang Academy of Agricultural Sciences in 2020 and 2021 using a completely randomized design with two replications. Each accession was sown in four plots on two columns of 1.5 m-wide beds, with 40 cm inter-row spacing and 75 cm individual spacing. Normal field management was used for plant growth and development over the 2 years.

For each accession, at least 10 representative immature pods were collected for pod size evaluation. These pods were grown for about 15 days after pollination, which was when they reached the commercial maturity stage and could be used only as vegetables. The PL was measured using a ruler, PW and PT were measured on the middle section of pods using a vernier caliper, and the single pod weight (SPW) was measured using a scale. The phenotype data of two replications on each trait were averaged. The data analyses, including frequency distributions, correlation analyses, and variance analyses, were conducted using Origin software (Version 2018). Multivariate analysis of variance (MANOVA) was conducted using SPSS software (v.22) under a general linear model (GLM).

### Genotyping and population structure analysis

The genotype data of the 88 accessions were retrieved from the study by Wu et al. (2022). What follows is a brief summary of the procedure. All accessions were sequenced with 12 × genome coverage using Illumina re-sequencing, 652.97 Gb of data were generated, and the clean reads were aligned to the common bean reference genome *P. vulgaris* v2.1 (https://phytozome-next.jgi.doe.gov/info/Pvulgaris_v2_1) using Burrows-Wheeler Aligner (BWA) software (http://bio-bwa.sourceforge.net/). Subsequently, the aligned files were converted to binary alignment map (BAM) files using SAMtools software, and, finally, the valid BAM files were used for SNP calling using the genome analysis toolkit (GATK) “UnifiedGenotyper” function (http://www.broadinstitute.org/gatk/). A total of 20,175,784 SNPs were identified. The criteria of a missing call rate > 0.5 and minor allele frequency (MAF) < 0.05 were used to filter the original SNPs. Finally, 603,910 high-quality SNPs were retained for population structural analysis and GWAS.

### GWAS

To detect the genomic regions associated with pod size, a GWAS was conducted on each trait using Tassel (v5.2.82) under a compressed mixed linear model (MLM) that accounted for population structure. The genetic effect of each SNP to the total phenotypic variation was calculated based on the marker *R* ^2^ values. The SNPs showing logarithm of the odds (LOD) values of ≥3.5 or 6.0 were regarded as significant SNPs. If two significant SNPs were located in the same linkage disequilibrium (LD) block (Delfini et al., 2021b; Valdisser et al., 2017; Wu et al., 2019), they were considered to represent the same QTL.

### Synteny analysis of pod size QTLs

The reported pod size QTLs/genes in common bean were first identified through a literature search, and then the sequences of the linked markers to these genes were blasted against the common bean reference genome *P. vulgaris* v2.1 (https://phytozome-next.jgi.doe.gov/info/Pvulgaris_v2_1) to determine their physical position under an e-value cut-off of 1e^−10^. By comparing the physical positions of the known pod size QTLs/genes and new QTLs detected in this study, their syntenic relations were determined, and if only two QTLs/genes were located in a LD block they were considered to represent a single gene/locus.

### Candidate genes mining and gene expression analysis

According to the estimated LD decay distance for common bean (Delfini et al., 2021b; Valdisser et al., 2017; Wu et al., 2019), the potential candidate genes residing in the 100 kb upstream and downstream of each SNP locus were investigated in the *P. vulgaris* v2.1 genome. Based on their annotation information, the genes with functions in developmental processes were considered as possible candidate genes. In addition, the gene expression data in different tissues were retrieved from *P. vulgaris* v2.1 and analyzed using Tbtools software (v1.098).

### KASP markers development and validation

To facilitate the molecular breeding of pod size in the common bean, the sequence of the significant SNP was used to design kompetitive allele-specific polymerase chain reaction (KASP) markers using a local script. The KASP genotyping for the 88 accessions was performed on an IntelliQube workstation (LGC Genomics Ltd., Hoddesdon, UK) following the PCR process described in the study by Wu et al. (2021). The haplotype analysis was conducted using Origin software (Version 2018).

## Results

### Phenotypic variation of pod size traits

**Figure 1:**
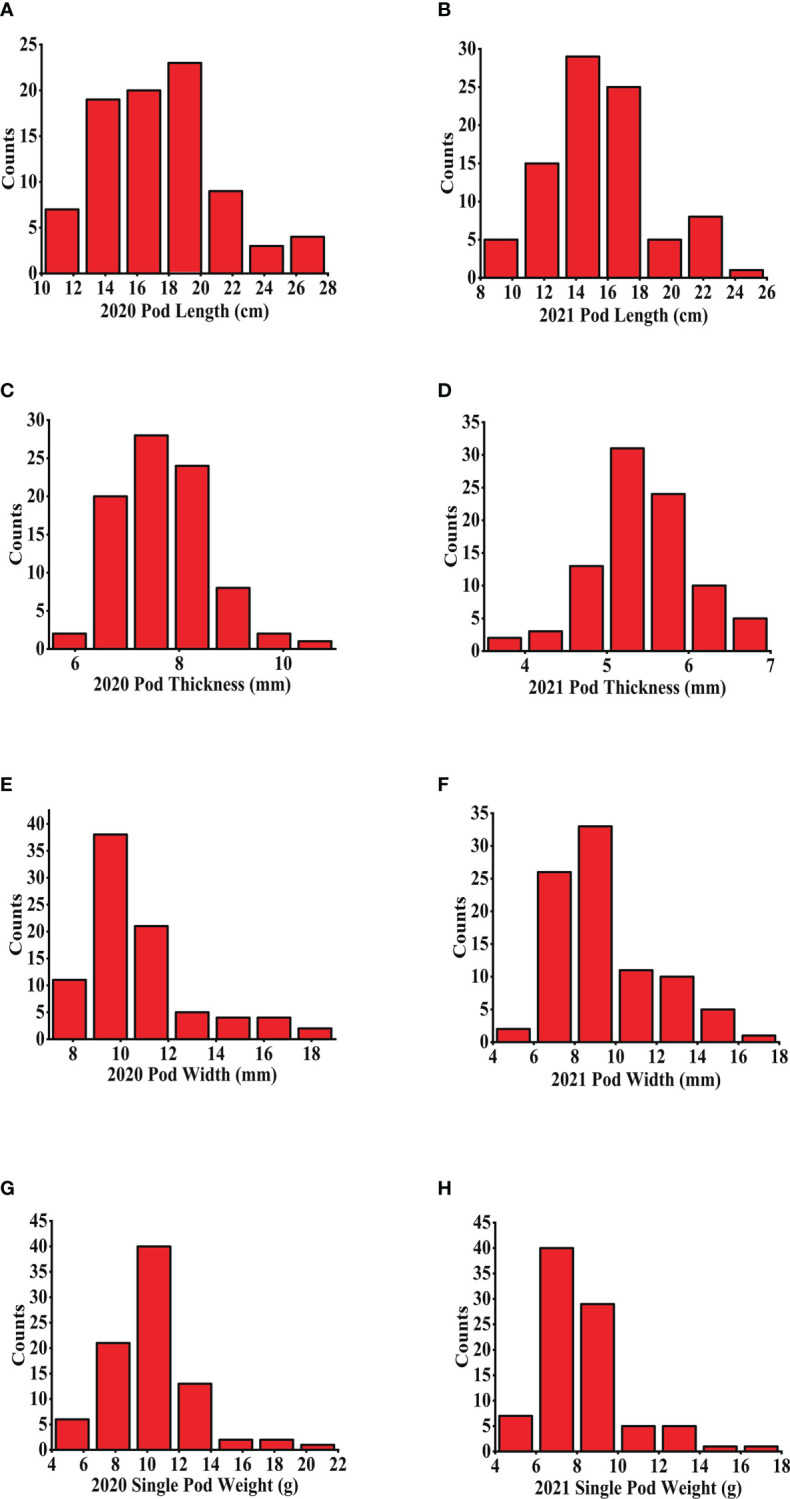
The frequency distribution of pod size over the 2 years. **(A, B)** PLs in 2020 and 2021, respectively; **(C, D)** PTs in 2020 and 2021, respectively; **(E, F)** PWs in 2020 and 2021, respectively; **(G, H)** SPWs in 2020 and 2021, respectively. PL, pod length; PT, pod thickness; SPW, single pod weight; PW, pow width.

**Figure 2:**
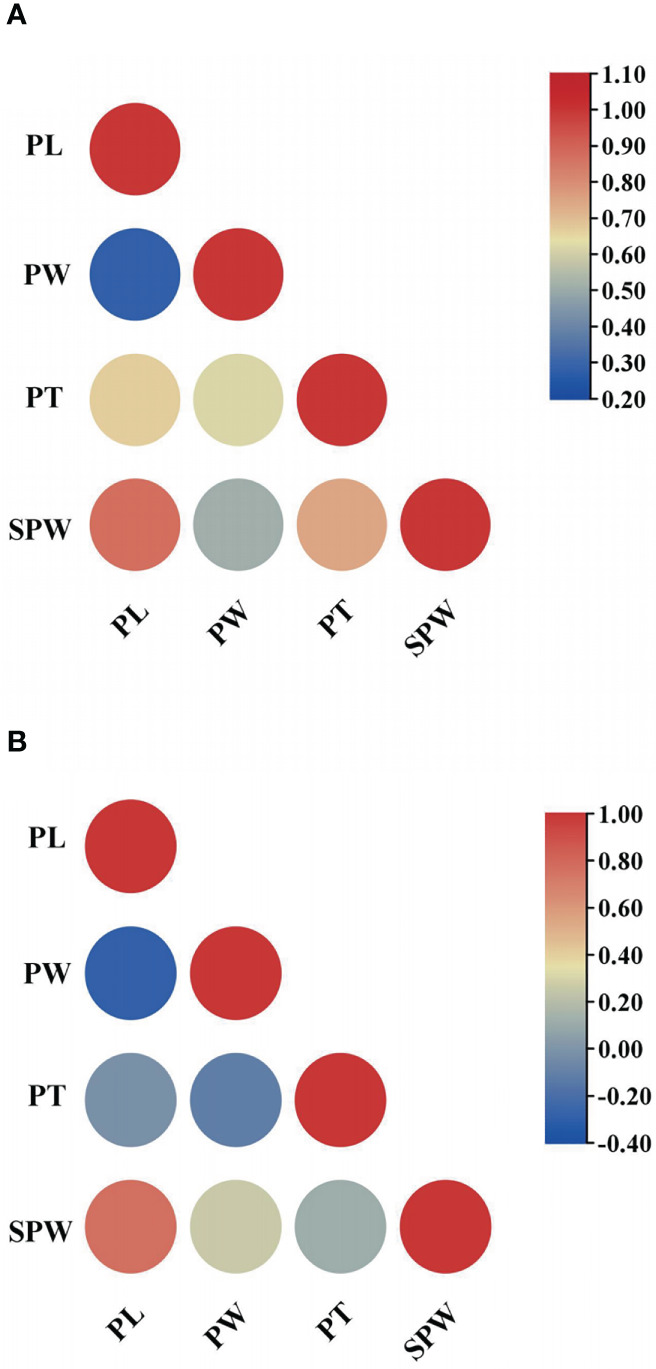
Correlations among pod size traits. **(A)** the correlation in 2020; **(B)** the correlation in 2021.

As shown in **Table 1** and **Figure 1**, the pod size showed a high level of variation in the 88 common bean accessions over the 2 years. The PLs ranged from 10.22–41.82 cm and 9.64–24.15 cm in 2020 and 2021, respectively. The PWs ranged from 7.61–18.64 mm and 5.22–17.18 mm in 2020 and 2021, respectively. The PTs ranged from 5.76 mm to 10.62 mm in 2020, and 3.72 mm to 6.86 mm in 2021. The SPWs also demonstrated continuous variation, ranging from 4.60–20.60 g in 2020 and 4.40–16.70 g in 2021. PL and PT showed the largest and smallest variance values by environmental changes in individual years, respectively, indicating that PL displayed the highest variation on the population level. The analysis of variance (ANOVA) statistics indicated that all four traits were affected significantly, by environmental changes in individual years with significant differences presenting among all genotypes, excluding those for PT, and significant interaction between genotypes and environments displayed for PL and PW (**Table S1**), indicating that it may be inappropriate to combine the two years data for further analysis. All four pod size traits displayed a nearly normal distribution in the diversity panel (**Figure 1**). Among the four traits, PL showed a higher positive correlation with both SPW (0.872) and PT (0.671), and a higher positive correlation (0.749) was also found between PT and SPW (**Figure 2**).

**Table 1:** The statistics of pod size traits in this study.

Trait according to year	Max	Min	Mean ± SE	Var	CV (%)
2021 PL (cm)	24.15	9.64	15.53 ± 0.35	10.99	21.35%
2020 PL (cm)	41.82	10.22	17.8 ± 0.54	24.39	27.75%
2021 PW (mm)	17.18	5.22	9.41 ± 0.27	6.43	26.97%
2020 PW (mm)	18.64	7.61	10.62 ± 0.26	5.72	22.53%
2021 PT (mm)	6.86	3.72	5.41 ± 0.06	0.36	11.21%
2020 PT (mm)	10.62	5.76	7.69 ± 0.09	0.76	11.34%
2021 SPW (g)	16.70	4.40	8.31 ± 0.23	4.72	26.17%
2020 SPW (g)	20.60	4.60	10.19 ± 0.29	7.03	26.04%

Max, maximum; Min, minimum; SE, standard error; Var, variance; CV, coefficient of variation; PL, pod length; PT, pod thickness; PW, pod width; SPW, single pod weight.

### Marker trait associations

**Figure 3:**
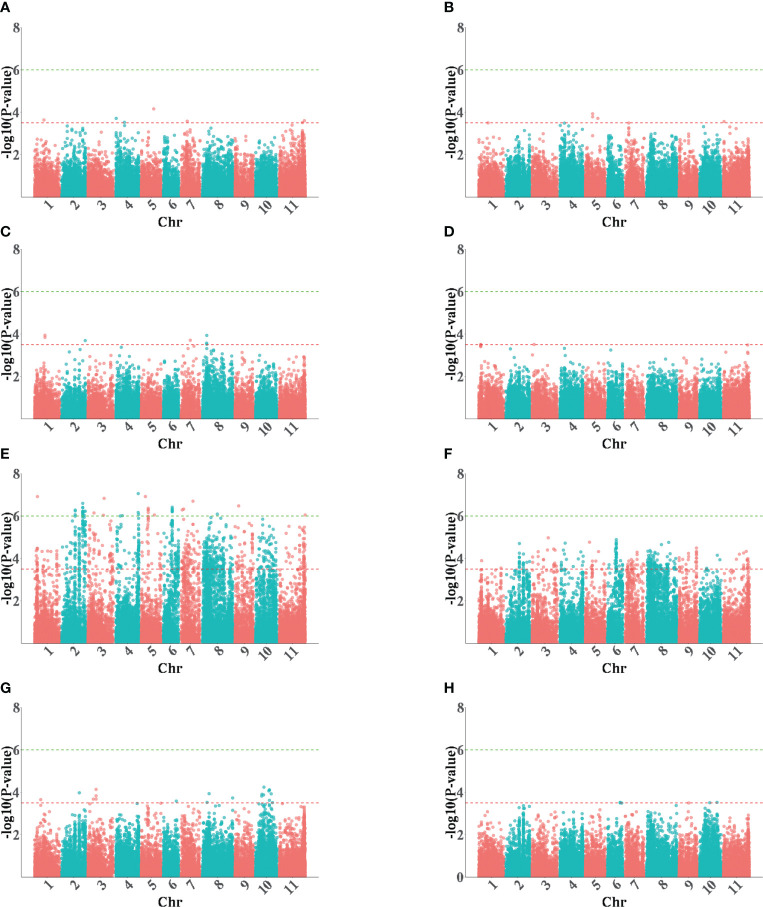
The Manhattan plots of GWAS for the pod size in 2020 (left) and 2021(right). **(A, B)** PLs; **(C, D)** PTs; **(E, F)** PWs; **(G, H)** SPWs. PL, pod length; PT, pod thickness; SPW, single pod weight; PW, pow width.

In a previous study, the 88 accessions were genotyped by re-sequencing and used for detecting SNPs significantly associated with rust resistance (Wu et al., 2022). The results of principal component analysis (PCA), neighbor-joining tree analysis, and structural analysis all supported that these accessions could be divided into two main clusters, which corresponded with the Middle American and Andean gene pools (Wu et al., 2022). To detect the pod size signals, a GWAS was conducted and a total of 1,241 SNPs with significant association with pod size were identified under the threshold value ≥ 3.5, which was distributed on all 11 chromosomes and accounted for 13.5%–59.1% of the phenotypic variation (**Figure 3**). It was also found that some SNPs formed clusters in different chromosomes and their adjacent distance ranged between 1 bp and hundreds of bps, indicating that some SNPs may represent a single pod size gene based on the 100 kb LD decay distance in common beans (Delfini et al., 2021b; Valdisser et al., 2017; Wu et al., 2019). After removing the redundant SNPs in an LD block and filtering the PW loci using a higher threshold value (≥ 6.0), a final 57 representative loci were reported (**Table 2**).

**Table 2:** The information of the detected pod size SNPs in current study.

Trait	SNP	Year(s)	Chr	Pos	LOD	Marker *R* ^2^
PL	Pv_0026128	2020	1	19213953	3.63	22.32%
	Pv_0285901	2020	4	344060	3.71	23.90%
	Pv_0311899	2020	4	17510650	3.53	13.47%
	Pv_0429726	2021	5	14767260	3.93	16.58%
	Pv_0445220	2020 and 2021	5	25626390	4.16	20.33%
	Pv_0562916	2020	7	13389784	3.58	18.23%
	Pv_1016502	2021	11	1129181	3.56	21.11%
	Pv_1129860	2020	11	47823838	3.51	19.17%
	Pv_1154801	2020	11	51850889	3.60	22.37%
PT	Pv_0002282	2021	1	3846586	3.51	27.14%
	Pv_0029912	2020	1	21338661	3.94	21.39%
	Pv_0201428	2020	2	48802744	3.69	29.50%
	Pv_0212827	2021	3	4133573	3.51	22.87%
	Pv_0579990	2020	7	19691399	3.71	24.30%
	Pv_0684832	2020	8	8785254	3.57	19.69%
	Pv_0685388	2020	8	9098191	3.93	30.82%
PW	Pv_0008234	2020 and 2021	1	5648221	6.92	47.27%
	Pv_0127220	2020 and 2021	2	28035744	6.28	37.42%
	Pv_0193604	2020	2	43687336	6.60	38.01%
	Pv_0195014	2020	2	46751740	6.22	38.70%
	Pv_0203965	2020	2	48895245	6.23	35.07%
	Pv_0226301	2020 and 2021	3	12533721	6.14	48.33%
	Pv_0272703	2020 and 2021	3	32138995	6.04	51.31%
	Pv_0274424	2020 and 2021	3	33692550	6.83	43.40%
	Pv_0298015	2020 and 2021	4	8968938	6.00	44.63%
	Pv_0303974	2020 and 2021	4	13113489	6.02	39.84%
	Pv_0366735	2020 and 2021	4	46263029	7.06	45.80%
	Pv_0368967	2020	4	47052667	6.07	42.89%
	Pv_0421055	2020 and 2021	5	8368043	6.92	55.70%
	Pv_0428704	2020	5	14219936	6.37	46.13%
	Pv_0447805	2020 and 2021	5	27146151	6.05	44.38%
	Pv_0519622	2020 and 2021	6	18274170	6.42	34.04%
	Pv_0545768	2020	7	3001451	6.29	41.86%
	Pv_0547992	2020 and 2021	7	5917854	6.33	47.38%
	Pv_0602824	2020	7	25300279	6.70	51.51%
	Pv_0721251	2020 and 2021	8	31136317	6.09	36.65%
	Pv_0802255	2020 and 2021	9	7474435	6.48	48.03%
	Pv_1169490	2020 and 2021	11	53574929	6.05	49.57%
SPW	Pv_0015964	2020	1	12473517	3.65	17.25%
	Pv_0165916	2020	2	36567260	3.98	30.29%
	Pv_0223474	2020	3	11173316	3.68	27.31%
	Pv_0237496	2020	3	16882167	4.14	28.93%
	Pv_0527678	2021	6	26312586	3.53	24.43%
	Pv_0527777	2020	6	26833487	3.59	23.74%
	Pv_0686810	2020	8	9704115	3.52	27.79%
	Pv_0692879	2020	8	13885583	3.93	32.25%
	Pv_0791404	2020	8	63047615	3.73	19.24%
	Pv_0932953	2020	10	12316708	3.82	23.97%
	Pv_0933835	2020	10	12783478	3.91	31.57%
	Pv_0939702	2020	10	16283834	3.88	28.21%
	Pv_0940934	2020	10	16955046	4.24	31.88%
	Pv_0948680	2021	10	20652263	3.51	38.28%
	Pv_0959635	2020	10	27212413	4.06	35.19%
	Pv_0961930	2020	10	28382540	4.11	32.87%
	Pv_0968910	2020	10	32052969	3.93	32.43%
	Pv_0974939	2020	10	34687854	3.51	23.43%
	Pv_0977521	2021	10	35814018	3.52	17.54%

LOD, logarithm of the odds; PL, pod length; PT, pod thickness; PW, pod width; SPW, single pod weight.

Of the significant SNPs found, nine were for PL, seven for PT, 22 for PW, and 19 for SPW (**Table 2**). The PL SNPs were distributed on five chromosomes (i.e., Pv01, Pv04, Pv05, Pv07, and Pv11) and accounted for 13.5%–23.9% of the phenotypic variation. The PW signals were from all 11 chromosomes, excluding chromosome 10, and accounted for 34.0%–55.7% of the phenotypic variation. The PT SNPs were located on chromosomes Pv01, Pv02, Pv03, Pv07, and Pv08, and accounted for 19.7%–30.8% of the phenotypic variation. The SPW SNPs were located on six chromosomes (i.e., Pv01, Pv02, Pv03, Pv06, Pv08, and Pv10), and accounted for 17.3%–38.3% of the phenotypic variation. A total of 16 significant SNPs were detected in both environments, and 15 were PW SNPs (**Table 2**), indicating that PW has greater heredity and was less affected by the environments.

### Candidate genes analysis

A total of 618 predicated gene models were identified as the candidates for the 57 pod size SNPs, which belonged to multiple gene families, such as the cytochrome P450 protein family, major facilitator protein superfamily, and the MYB transcription factor family (**Table S2**). Among them, 26 gene models with annotated functions highly associated with plant growth and the development of 16 significant SNPs were identified (**Table 3**). Of which, 13 were cytochrome P450 proteins gene models which involve in the regulation of plant hormone metabolism and play important roles in plant growth and development (Xu et al., 2015). Among these 13 cytochrome P450 protein gene models, six (*Phvul.004G005700*- *Phvul.004G006100*) formed a cluster from 368,531 bp to 403,445 bp on chromosome Pv04, which has a 24.471 kb distance to the PL SNP Pv_0285901. We also identified two MYB transcription factor family gene models (*Phvul.008G094800, Phvul.010G075500*) and three WRKY protein gene models (*Phvul.008G090300, Phvul.002G265400, Phvul.002G266400*) that play crucial regulatory roles in plant developmental processes (Dubos et al., 2010; Wang et al., 2021). In addition, we identified other gene models, i.e., *Phvul.011G015300*, *Phvul.008G088100*, *Phvul.003G128800*, *Phvul.004G166000*, *Phvul.006G157800*, and *Phvul.006G159300*, involved in plant hormone biosynthesis and regulation that play a central role in controlling plant growth and development (Cui et al., 2010; Feng et al., 2013; Nibau et al., 2013; Zhao et al., 2015; Salem et al., 2017; Powers et al., 2019; Jing et al., 2022). The glycosyltransferase gene model *Phvul.005G091200* was proposed to be involved in secondary cell wall glucuronoxylan and/or pectin biosynthesis that increasing the stem growth in Populus (Biswal et al., 2015). The gene model *Phvul.003G072200* encodes an amino phospholipid ATPase, which complex forms an important part of the Golgi machinery required for secretory processes during plant development (Poulsen et al., 2008).

**Table 3:** Candidate genes for the detected pod size SNPs.

Trait	SNP	Pos	Gene	start	end	Annotated function
PL	Pv_0285901	344060	Phvul.004G005400	315927	317696	cytochrome P450, family 96, subfamily A, polypeptide 10
			Phvul.004G005700	368531	370087	cytochrome P450, family 96, subfamily A, polypeptide 10
			Phvul.004G005800	372078	374079	cytochrome P450, family 96, subfamily A, polypeptide 10
			Phvul.004G005900	380970	382499	cytochrome P450, family 96, subfamily A, polypeptide 10
			Phvul.004G006000	386618	394940	cytochrome P450, family 96, subfamily A, polypeptide 10
			Phvul.004G006050	394161	395837	cytochrome P450, family 96, subfamily A, polypeptide 10
			Phvul.004G006100	401756	403445	cytochrome P450, family 96, subfamily A, polypeptide 10
			Phvul.004G006300	431954	433623	cytochrome P450, family 96, subfamily A, polypeptide 1
	Pv_0445220	25626390	Phvul.005G091200	25636647	25640316	galacturonosyltransferase 12
	Pv_1016502	1129181	Phvul.011G015300	1205296	1211056	P-loop containing nucleoside triphosphate hydrolases superfamily protein
PT	Pv_0684832	8785254	Phvul.008G088100	8689853	8700154	HEAT repeat; WD domain, G-beta repeat protein protein
	Pv_0685388	9098191	Phvul.008G090300	9078509	9081654	WRKY DNA-binding protein 33
PW	Pv_0008234	5648221	Phvul.001G052100	5695145	5699567	cytochrome P450, family 71, subfamily B, polypeptide 35
	Pv_0193604	43687336	Phvul.002G265400	43645971	43647134	WRKY DNA-binding protein 51
			Phvul.002G266400	43771606	43773653	WRKY DNA-binding protein 13
	Pv_0272703	32138995	Phvul.003G128800	32230627	32238415	auxin response factor 19
	Pv_0303974	13113489	Phvul.004G076200	13119224	13119583	cytochrome P450, family 72, subfamily A, polypeptide 15
	Pv_0366735	46263029	Phvul.004G159300	46348261	46352994	cytochrome P450, family 71, subfamily B, polypeptide 34
			Phvul.004G159500	46359615	46361846	cytochrome P450, family 71, subfamily B, polypeptide 34
	Pv_0368967	47052667	Phvul.004G166000	46991847	46995082	RHO-related protein from plants 9
SPW	Pv_0223474	11173316	Phvul.003G072200	11117010	11145109	aminophospholipid ATPase 3
	Pv_0237496	16882167	Phvul.003G085400	16950726	16952504	cytochrome P450, family 71, subfamily B, polypeptide 34
	Pv_0527678	26312586	Phvul.006G157800	26263616	26264925	Adenosylmethionine decarboxylase family protein
			Phvul.006G159300	26336534	26342306	histidine-containing phosphotransmitter 1
	Pv_0686810	9704115	Phvul.008G094800	9681839	9684032	myb-like HTH transcriptional regulator family protein
	Pv_0961930	28382540	Phvul.010G075500	28283209	28293553	myb domain protein 1

SNP, single nucleotide polymorphism; PL, pod length; PT, pod thickness; PW, pod width; SPW, single pod weight.

**Figure 4:**
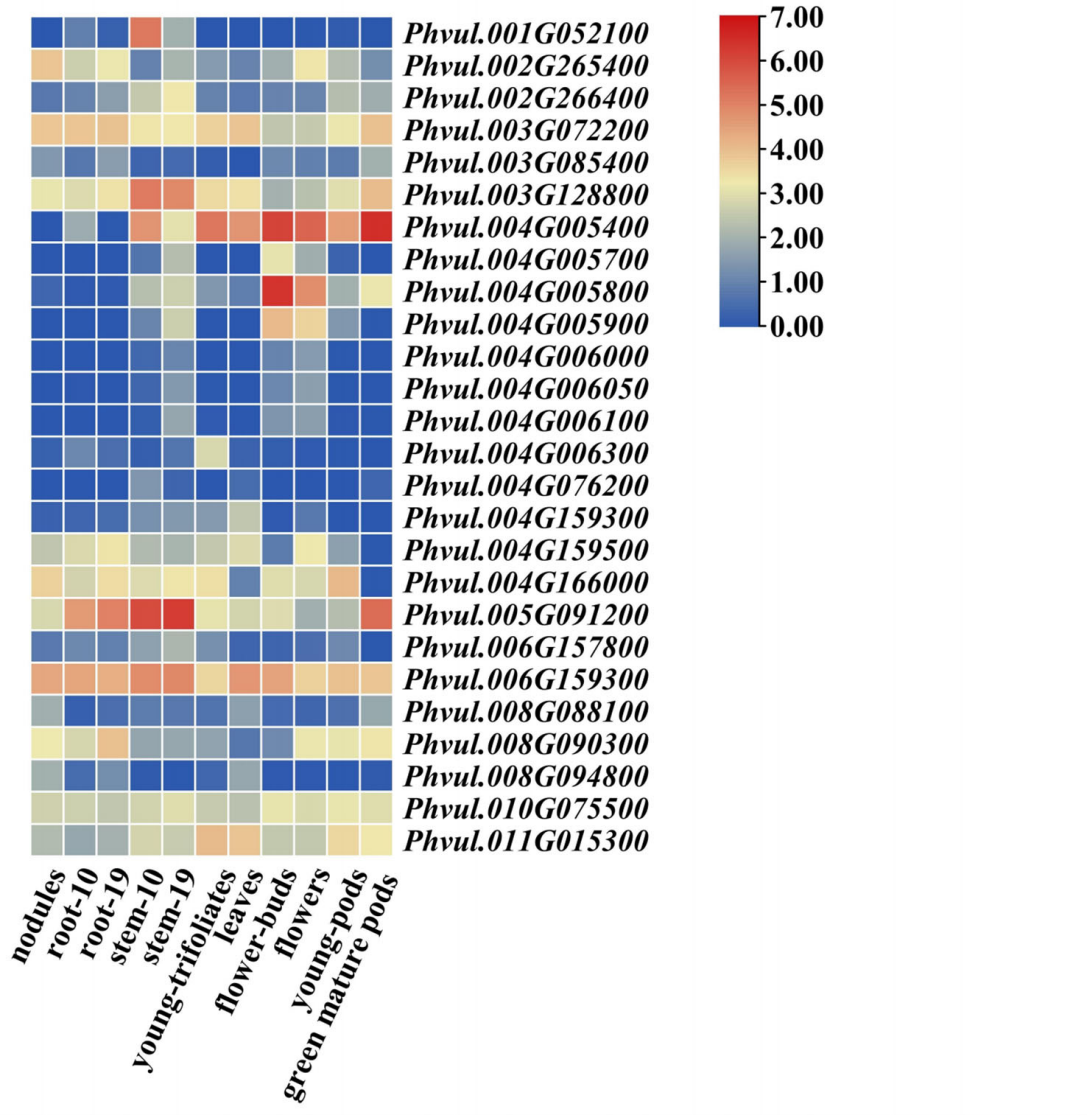
Expression patterns of 26 candidate genes in 11 tissues of common bean.

The results of an *in silico* analysis showed that 26 candidate gene models demonstrated a significantly different expression pattern in different tissues, and eight genes were found to be more abundantly expressed in flower buds, flowers, young pods, and green mature pods, indicating that they may be involved in the pathways of pod development (**Figure 4**). The genes with higher expression levels in young pods included the amino phospholipid ATPase family protein gene (*Phvul.003G072200*), the auxin response factor (*Phvul.003G128800*), cytochrome P450 gene (*Phvul.04G005400*, *Phvul.04G005800*), the hormone biosynthesis and regulation genes (*Phvul.011G015300* and *Phvul.006G159300*), the MYB transcription factor *Phvul.010G075500* and the WRKY gene *Phvul.008G090300*, suggesting that hormonal and nutritional signaling are also important mechanisms related to pod size control.

### Development of KASP markers for the important loci

**Figure 5:**
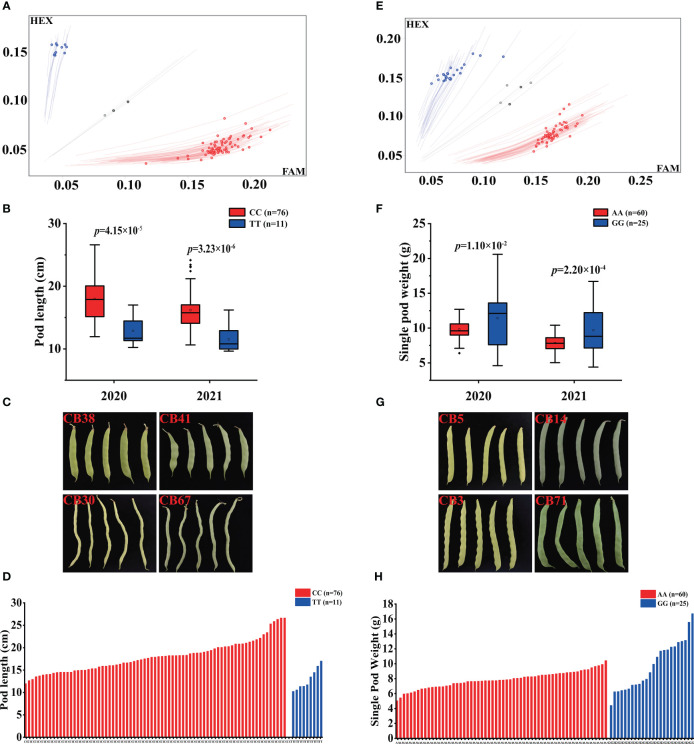
KASP markers development for PL SNP Pv_0026128 and SPW SNP Pv_974939. **(A)** genotype signal for KASP Pv_0026128 marker; **(B)** haplotypes analysis of Pv_0026128 in the diversity panel; **(C)** pods of representative accessions with different haplotypes on Pv_0026128; **(D)** the PLs and their genotype of each accession in the panel; **(E)** genotype signal for KASP Pv_974939 marker; **(F)** haplotypes analysis of Pv_974939 in the diversity panel; **(G)** pods of representative accessions with different haplotypes on Pv_974939; **(H)** the SPWs and their genotype of each accession in the panel. GWAS, genome-wide association study; KASP, kompetitive allele specific polymerase chain reaction; PL, pod length; SNP, single nucleotide polymorphism; SPW, single pod weight.

To facilitate the molecular breeding of pod size, a PL SNP Pv_0026128 and a SPW SNP Pv_974939 were selected for conversion into PCR-based KASP markers (**Table 4**) and were successfully validated in the diversity panel (**Figures 5A, E**). The Pv_0026128 showed *CC* and *TT* genotypes for 88 accessions, and the haplotype Pv_0026128-CC showed a longer PL (**Figures 5B–D**; the average PL is 17.96 cm), which could increase the PL by 5.08 cm. The Pv_974939 displayed an AA and a GG haplotype with 60 and 25 accessions, respectively. The haplotype Pv_974939-GG had more SPW than that of Pv_974939-AA (**Figures 5F–H**; the average SPW is 9.68 g), which increased the SPW by 1.84 g. These results indicate that the two markers could be used for PL and SPW selection in snap bean breeding.

**Table 4:** The primer sequence information of the two KASP markers.

SNP	Primer_AlleleFAM	Primer_AlleleHEX	Primer_Common	Allele FAM	Allele HEX
Pv_0026128	GAAGGTGACCAAGTTCATG CTAGTTGCTTCAACCTGGAGC	GAAGGTCGGAGTCAACGGATT AGTTGCTTCAACCTGGAGT	TAAGTAATTGTTAT CGCTTGCTGACCT	C	T
Pv_0974939	GAAGGTGACCAAGTTCATGC TTCTGAGTCGTCTCGGGCACAA	GAAGGTCGGAGTCAACGGAT TTCTGAGTCGTCTCGGGCACAG	CTTGAGCGTCGGAGTGCCTTCT	A	G

KASP, kompetitive allele specific polymerase chain reaction; SNP, single nucleotide polymorphism.

## Discussion

Snap bean is the most commonly grown vegetable legume for human consumption. It is believed that snap beans are dry beans that were selectively bred after the Columbian Exchange and that have since been subject to intense selective breeding separately in Europe and China, the purpose of which has been to enhance such traits as low pod wall fiber and thick pod walls (Myers and Baggett, 1999; Zhang et al., 2008; Wallace et al., 2018). Indeed, pod size has long been of interest to breeders because it is an important yield and appearance quality trait. The improvement of pod size in snap bean requires a fundamental understanding of the genetic basis of pod size, and the associations between other pod morphological traits and QTL/genes have been identified in studies of foreign bean germplasms (Yuste-Lisbona et al., 2014; González et al., 2016; Hagerty et al., 2016; Gupta et al., 2020; Murube et al., 2020; García-Fernández et al., 2021). However, even though China is the largest snap bean producer in the world, investigations into the genetic architecture of pod size in Chinese snap bean germplasms are lacking. In the present study, a diversity panel consisting of 88 Chinese snap bean germplasms that has previously been used to identify rust resistance signals (Wu et al., 2022) was evaluated for pod size and a total of 57 SNPs significantly associated with pod size were detected using GWAS. The panel size is relatively small; however, population structure analysis showed that it could be divided into the Andean and Middle American gene pools that are well established in common bean, a finding that is consistent with the population division of a larger Chinese common bean in which 683 accessions were identified (Wu et al., 2019). In addition, pod size traits such as PL and PW showed higher levels of heritability (0.89 and 0.91, respectively) (García-Fernández et al., 2021), indicating the stronger resolution of these traits for gene mapping. Therefore, based on the above two conditions, we deemed it practicable to use a small panel to investigate the genetic architecture related to pod size. Among this panel, 12 accessions belonged to the Andean gene pool; this ratio is lower than that of the American snap bean germplasms, in which most snap bean accessions belonged to the Andean gene pool (Song et al., 2015). This is not surprising given that China is the secondary center of diversity for common bean (Zhang et al., 2008), and snap bean has been widely cultivated in diverse agro-ecosystems, with environments ranging from subtropical to temperate and from sea level to 3,000 meters above sea level (masl). Therefore, snap beans were possibly selectively bred from both the Andean and Middle American gene pools by people in diverse areas, and natural or artificial inter-gene pool introgression may have also led to this shift. In fact, previous studies have suggested that snap beans may have originated from more than one gene pool (Wallace et al., 2018). In any case, our results indicate the optimal genes and phenotypes that could be used in breeding lines, especially in China.

The LD decay distance varied in different common bean collections using different Brazilian common bean diversity panels. Valdisser et al. (2017) found that the LD extension estimate was 395 kb for the Andean gene pool and 130 kb for the Middle American gene pool, and Delfini et al., (2021b) calculated that the LD decay value is 296 kb. Using a Chinese common bean diversity panel consisting of 683 accessions, Wu et al. (2019) found that the average LD decay distance between SNPs was 107 kb across the genome. In our previous study, the LD decay distance was found to be 800 kb (Wu et al., 2022). Because of the relatively small population size in this study, however, we regarded an LD decay distance of 800 kb as being unrepresentative. Considering that all the 88 accessions belonged to Chinese landraces and cultivars, which may bear a much closer resemblance to the diversity panel used in a previous study by Wu et al. (2019), we finally decided on a decay distance of ± 100 kb LD to identify the overlapped SNPs and search the candidate gene models for each significant SNP.

According to the ±100 kb LD, it was found that a PT SNP Pv_0201428 (chr02: 48,802,744) has a 92.501 kb distance to the PW SNP Pv_0203965 (chr02: 48,895,245), indicating that they may represent a single locus. This pleiotropic effect on PW and PT was also investigated in a previous study, in which a pod width QTL PWI4 and a pod thickness QTL PT4.1 were mapped on the same interval (Yuste-Lisbona et al., 2014). We also found that these two SNPs were located in a QTLs enrichment interval from 48,634,684 bp to 49,605,168 bp on chromosome 2, where five pod-related QTLs including a pod color QTL (PodLCol2_49.4), a PL QTL (PL2.2XC), two numbers of seeds per pod QTLs (NSP02_48.7 and NSP2XC), and a pod perimeter QTL (E-PP2XB) (Murube et al., 2020; García-Fernández et al., 2021), were detected. In addition, we found that a PW SNP Pv_0519622 and a SPW SNP Pv_0237496 were located in the intervals of the PW QTL PWI6 and the QTL NSP3 for the number of seeds per pod (Murube et al., 2020), respectively, suggesting that pod morphological trait QTLs often demonstrate pleiotropic effects, or that they are clustered, or closely linked, in the genome.

As a fruit organ, pod development is a complex process that is regulated by plant hormones and transcription factors (Tang et al., 2020). Cytochrome P450 family genes WRKY and MYB transcription factors were the predominant candidate genes for pod size SNPs detected in this study, which were also the main candidates for pod morphological QTLs in previous study (García-Fernández et al., 2021). For the PL SNP Pv_0285901, a total of eight cytochrome P450 genes were identified as its candidates, six of which formed a cluster and two genes, *Phvul.004G005400* and *Phvul.004G005800*, showed higher expression levels in flowers and young pods (**Table 3**; **Figure 4**). The cytochrome P450 genes *BnaC7.ROT3* and *BnaA9.CYP78A9* both mediate silique length by affecting cell elongation in rapeseed through the unknown auxin biosynthesis pathway (Shi et al., 2019; Zhou et al., 2022). Thus, it is suggested that these two genes were highly related to the gene function of this locus and further investigation was needed. A P-loop containing nucleoside triphosphate hydrolases superfamily protein (*NOA1*) *Phvul.011G015300* was considered as a candidate gene of the *PL SNP Pv_1016502*, which is involved in growth regulation and hormonal signaling in plants (Zhao et al., 2015). The auxin response factor *Phvul.003G128800* is regarded as the candidate gene for the PW SNP Pv_0272703, whereas auxin response factor 18 (*ARF18*) affects the seed weight and silique length by regulating cell growth in polyploid rapeseed (Liu et al., 2015). In addition, we found that a total of 19 candidate gene models are involved in the biosynthesis and regulation of plant hormones, indicating that auxin-response pathways play key roles in pod size development in snap beans, which indicates clear pathways for the cloning of pod size genes in future.

In summary, we detected 57 genomic regions that were significantly associated with pod size in Chinese snap bean germplasms, which provides genomic resources for molecular breeding of pod size. The two developed KASP markers could be used directly in the marker-assisted selection of pod size traits. The results also enhanced our understanding of the molecular mechanism of pod development and established a springboard for future pod size gene cloning and its application in snap bean cultivation.

## Data availability statement

All sequencing data of the 88 bean accessions are available from NCBI under BioProject accessions no. PRJNA934100.

## Author contributions

XYW, GL, and JY conceived and designed the research; ML performed the experiments, analyzed the data, and wrote the draft manuscript; BW, XHW, and ZL contributed to germplasm collection; YW, JWa, YS, and WD contributed to phenotype evaluation. JD and JWu helped analyzing the data. XYW analyzed the data and revised the manuscript. All authors contributed to the article and approved the submitted version.
